# Characterization of Parameters Required for Effective Use of Tamoxifen-Regulated Recombination

**DOI:** 10.1371/journal.pone.0003264

**Published:** 2008-09-23

**Authors:** Ben Buelow, Andrew M. Scharenberg

**Affiliations:** Departments of Pediatrics and Immunology, University of Washington, Seattle Children's Hospital Research Institute, Seattle, Washington, United States of America; National Institute on Aging, United States of America

## Abstract

Conditional gene targeting using the Cre-loxp system is a well established technique in numerous *in vitro* and *in vivo* systems. Ligand regulated forms of Cre have been increasingly used in these applications in order to gain temporal and spatial control over conditional targeting. The tamoxifen-regulated Cre variant mer-Cre-mer (mCrem) is widely utilized because of its reputation for tight regulation in the absence of its tamoxifen ligand. In the DT40 chicken B cell line, we generated an mCrem-based reversible switch for conditional regulation of a transgene, and in contrast with previous work, observed significant constitutive activity of mCrem. This prompted us to use our system for analysis of the parameters governing tamoxifen-regulated mCrem recombination of a genomic target. We find that robust mCrem expression correlates with a high level of tamoxifen-independent Cre activity, while clones expressing mCrem at the limit of western blot detection exhibit extremely tight regulation. We also observe time and dose-dependent effects on mCrem activity which suggest limitations on the use of conditional targeting approaches for applications which require tight temporal coordination of Cre action within a cell population.

## Introduction

Conditional gene targeting allows for spatial and temporal control of gene expression both *in vitro* and *in vivo*. Several systems for conditional gene targeting exist, such as Flp/Frt, Cre/Loxp, ΦC31-att, Mx-1/IFN-l·α, and Tet on/off [1], [2]. One of the most popular is the Cre-loxp system: a medline search for “Cre recombinase” yields over 2000 publications, of which about 900 represent transgenic mice [3]. For this approach, a portion of a gene is flanked by loxp sites, 34 base pair sequences consisting of two 13 base inverted repeats flanking 8 non-palindromic bases. Cre recombinase, nominally specific for these sites [4], [5], either excises or inverts flanked sequences depending on the relative orientation of the two loxp sites, leading to changes in gene function and/or expression. Historically, the popularity of the Cre/loxp system derives from its apparent high fidelity, absence of canonical recognition sites in the mammalian genome, and its high efficiency of recombination as compared to other strategies [6], [7].

Most of the work using the Cre-loxp system has been predicated on the assumption that Cre, without Loxp targets, is largely inactive in vertebrate cells. This assumption may have arisen in part due to the lack of overt pathology in Cre expressing mice [8], but given the ability of mammals to tolerate significant somatic cell death this conclusion clearly requires validation. Several studies, highlighted recently in two articles [3], [9] have shown that use of the Cre-loxp system in eukaryotic cells carries with it numerous potential confounders such as variable efficiency of Cre expression and/or recombination, variability in germline recombination, and, contrary to the previously mentioned assumption, Cre mediated toxicity [10]–[14].

In order to gain temporal and spatial control over conditional gene targeting, ligand regulated forms of Cre are often utilized. The tamoxifen-regulated Cre variants Cre-ER^T^ (Cre fused to a mutated human estrogen receptor that binds tamoxifen or 4-hydroxytamoxifen (OHT) but not endogenous estrogens, [11], [15]–[18]), and mer-Cre-mer (mCrem, which fuses Cre to two mutated murine estrogen receptors responsive to tamoxifen/OHT but not endogenous estrogens, much like Cre-ER^T^ above [19], [20]) are favored ligand-regulated Cres because of their reputation for tight regulation in the absence of their tamoxifen/OHT ligands [20]–[25]. Tamoxifen/OHT-regulatable Cre fusion proteins demonstrate titratable toxicity, suggesting that Cre mediated toxicity is dose- and exposure time-dependent. Over the course of these titration studies it was observed that even in the absence of the inducing ligand some cytotoxicity and/or gene expression could be seen in several cell systems, including DT40 B lymphocytes [11], [19], [26], [27]. Additionally, it has been suggested that ligand independent effects of inducible Cre may be altered by the expression level of the Cre protein [26]. These results call the efficiency of regulation of these Cre variants into question, and suggest a need for a better understanding of how the parameters of inducible Cre expression, Tamoxifen/OHT dose, and Tamoxifen/OHT time of exposure interact to affect tight regulation of ligand-induced Cre activity.

**Figure 1:**
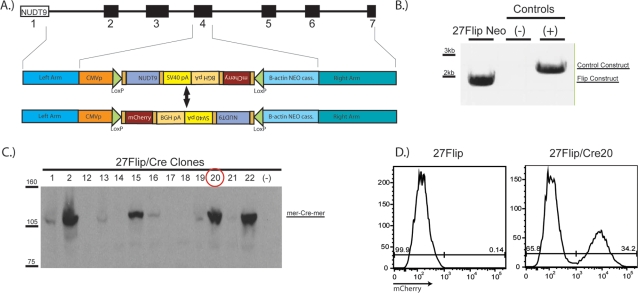
DT40 B lymphocytes transfected with a reversible switching construct and expressing mer-Cre-mer show 4-hydroxytamoxifen independent Cre activity. *A* Schematic of the “Flip” construct. Exon 4 of NUDT9 was replaced with a CMV promoter, an in-frame region (containing a NUDT9 cDNA and an inverted mCherry cDNA, each followed by a poly-A region) flanked by loxp sites, and a neomycin selection cassette. Treatment of cells stably expressing the construct with 4-hydroxytamoxifen (OHT) should lead to reversible flipping of the floxed region, as shown in the cartoon, and subsequent expression of mCherry red fluorescent protein in ∼50% of the cells. *B* Stable integration of the Flip construct was verified in clone #27 via genomic PCR. The positive control represents a different construct that could be amplified with the same primer set. *C* Following stable transfection of clone #27 with a mer-Cre-mer (mCrem) expressing vector, cells from various clones were lysed and 50 µg of protein from each were run on an 8% SDS-PAGE gel. mCrem was visualized with polyclonal rabbit anti-Cre antibody (1∶2000, Novagen), and a highly expressing clone, 27Flip/Cre20 was selected for further study (red circle). *D* 27Flip and 27Flip/Cre20 cells were analyzed by flow cytometry, as indicated. In contrast to the 27Flip parental line, the mCrem expressing 27Flip/Cre20 cells showed robust red fluorescence in a large subpopulation despite the absence of the mer- ligand OHT.

Our lab makes extensive use of DT40 B-cells as a model system because they support facile gene targeting and are well validated for studies of lymphocyte signaling and physiology [28]–[34]. To effect conditional gene targeting in these cells, we have historically used mCrem [35]. In the process of generating a Cre-mediated reversible switching line with a fluorescent readout of Cre activity (Fig. 1A), we observed a high level of ligand-independent mCrem activity. To understand how best to implement mCrem-based conditional gene targeting, we have used this system to explore the parameters governing tamoxifen-induced mCrem activity. The results of our investigation show that the level of mCrem expression correlates with Cre activity independent of OHT treatment: high expression leads to abundant ligand-independent activity, while low mCrem expression essentially abolishes activity. Evaluation of time and dose effects in clones with variable expression of mCrem suggests important limitations on the use of mCrem for applications requiring temporally correlated Cre activity within cell populations.

## Results

### DT40 B lymphocytes transfected with a reversible switching construct and expressing mer-Cre-mer show OHT-independent inversion of the floxed cassette

To better understand the function of the Nudix-type ADPR-hydrolase NUDT9, our lab targeted this gene for deletion in DT40 chicken B cells. Since we were unable to target both alleles with classical knockout constructs, we reasoned that loss of NUDT9 must be lethal in this cell type, and that therefore a conditional knockout strategy would be required. We chose to proceed with a reversible switching construct (Fig. 1A), in which a chicken NUDT9 cDNA and an mCherry fluorescence reporter were present in inverted orientation to one another. This organization afforded us a fluorescent readout of Cre activity, with activity in 100% of cells correlating to roughly 50% red fluorescent cells [24]. Following selection of a clone that had stably integrated the construct (Fig. 1B), we transfected this clone, designated 27Flip, with a vector constitutively expressing mCrem under the control of the CMV promoter.

Following transfection of the 27Flip cells with the mCrem containing vector, stably expressing clones were identified and a highly expressing clone, designated 27Flip/Cre20 was selected on the basis of an mCrem western blot (Fig. 1C). Based on previous work [20], [24], it was anticipated that prior to treatment with OHT, 27Flip/Cre20 cells would show no mCherry fluorescence, similar to the parental 27Flip line. However, the 27Flip/Cre20 clone showed a sub population with robust red fluorescence (Fig. 1D) prior to any tamoxifen exposure. Thus, we sought to identify the cause of mCherry expression in these cells.

### Sustained OHT-independent flipping of the floxed cassette in 27Flip/Cre20 cells is a stable, ongoing process

**Figure 2:**
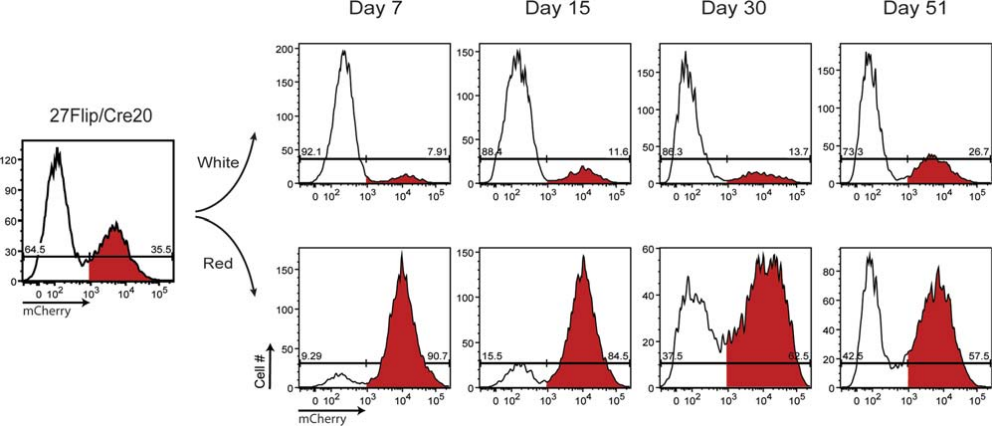
Sustained OHT-independent flipping of the floxed cassette in 27Flip/Cre20 cells is a stable, ongoing process. mCherry (+) and mCherry (−) 27Flip/Cre20 cells were separated by FACS and grown in culture for ∼7 weeks. During this time period, a steady decrease in the purity of the sorted populations was observed by flow cytometry. X-axis, mCherry fluorescence, Y-axis, cell number.

We initially hypothesized that electroporation or other stressors associated with transduction of DT40 cells with the mCrem containing construct might lead to inefficient exclusion of mCrem from the nucleus and subsequent Cre dependent switching of our construct in affected cells. To test this hypothesis, we sorted mCherry negative (“white”) and mCherry-positive (“red”) cells by flow cytometry, and monitored the fluorescence of the separated populations over time in the absence of OHT (Fig. 2). If mCrem activity were a transient event associated with electroporation, these sorted populations would be expected to remain stable over time. However, we observed that white cells showed an outgrowth of red cells and vice versa over a course of 7 weeks in culture. Interestingly, red cells converted to white cells more rapidly, a phenomenon potentially related to idiosyncratic differences in susceptibility of the flip cassette to recombination, or a survival disadvantage associated with loss of NUDT9 expression. These results suggested that in contrast to previous results, mCrem was capable of mediating significant levels of constitutive recombination activity. Because such unlicensed mCrem dependent recombination is a potential confounding factor for the reversible switching approach to conditional gene inactivation, we set out to better characterize this phenomenon.

### Sustained OHT-independent Cre activity in 27Flip/Cre20 cells correlates with mer-Cre-mer expression level

**Figure 3:**
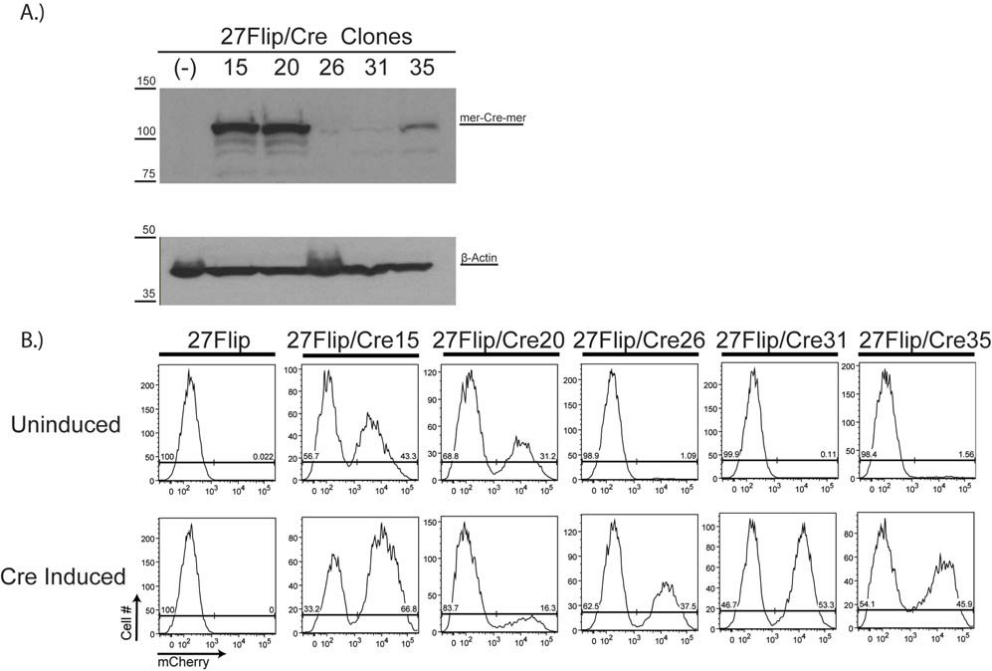
Sustained OHT-independent flipping of the floxed cassette in 27Flip/Cre20 cells correlates with mer-Cre-mer expression level. *A* Following stable transfection of 27Flip cells with an mCrem expressing vector, 5 clones expressing various amounts of mCrem were selected. Cells from the clones were lysed and 50 µg of protein of each were run on an 8% SDS-PAGE gel. MCrem was visualized with polyclonal rabbit anti-Cre antibody (1∶2000, Novagen), with β-actin visualized with a polyclonal mouse antibody (1∶40000, Sigma) as a loading control. 27Flip/Cre15 and -20 were designated “high expressors”, and 27Flip/Cre26, -31, and -35 were designated “low expressors”. *B* 27Flip/Cre clones were either left untreated or treated with 1 µM OHT for 48 hours and then allowed to grow in culture for 18 days: all clones were subsequently analyzed by flow cytometry. The 27Flip parental cell line was used as a negative control.

Several previous observations suggest that control of mCrem recombination is less than perfect [11], [20], [26], [27]. Based on these observations, we were interested in clarifying the role of mCrem expression level in OHT-independent recombination. We hypothesized that low frequency OHT-independent recombination by mCrem is mCrem intrinsic, and thus that OHT-independent Cre activity should be demonstrable in other mCrem expressing clones, and furthermore that as more mCrem would provide more activity, the level of constitutive flipping should correlate with mCrem expression level. To test this hypothesis, we re-transfected 27Flip cells with the mCrem containing vector to generate a panel of 27Flip/Cre clones expressing varying amounts of mCrem by western blot (Fig. 3A). These clones were assigned to either “high” (27Flip/Cre15 and -20) or “low” (27Flip/Cre26, -31, and -35) mCrem expressing categories and compared for flipping in the presence and absence of 1 µM OHT (Fig. 3B). Consistent with mCrem possessing intrinsic OHT-independent recombination activity, clones with high expression showed OHT-independent Cre activity, while low expressing clones exhibited little to no activity. Although the apparent decrease in flipping for the high expressing clones at high OHT concentrations suggested a potential difference in efficiency of recombination depending on the orientation of the cassette, repeating these experiments revealed variation in the size of the red population ranging from 38–60% that changed from day to day (data not shown), suggesting that background fluctuations in the population occur in the context of constitutive ongoing flipping. Importantly, all clones responded well to OHT treatment based on their establishing a ∼50∶50 ratio of red to white cells over a 48 hour OHT treatment (with a 50/50 ratio indicating flipping in ∼100% of cells). Note that clone 35, which at first glance appeared to have less red cells than other clones, showed poorer separation of red and white populations than other clones, making the percentage of white vs. red cells less precise in this clone than others.

### OHT-dependent mCrem activity saturates by 10 nM OHT

**Figure 4:**
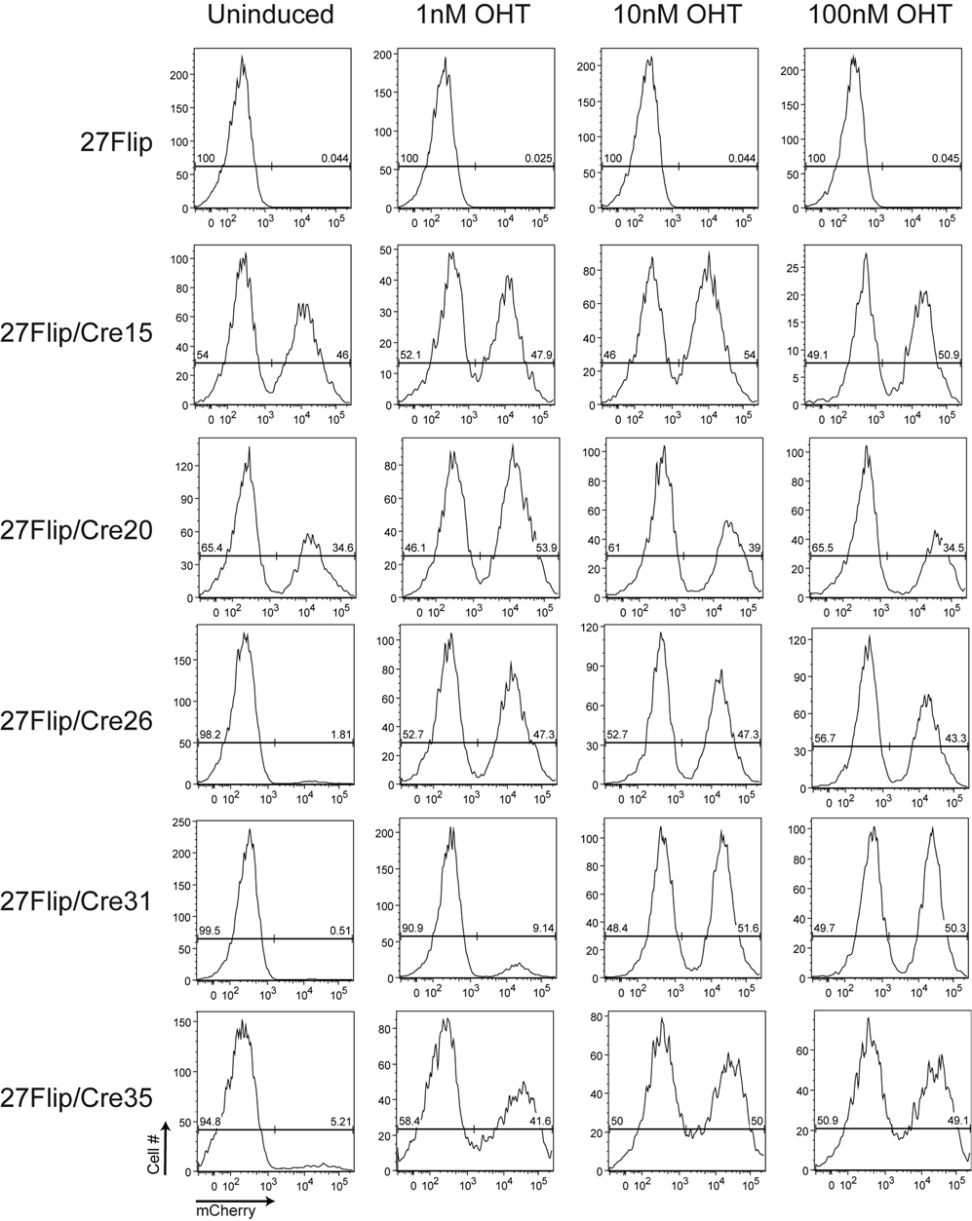
OHT-dependent flipping of the floxed cassette saturates by 10 nM OHT. 27Flip/Cre clones were either left untreated or treated with 1, 10, or 100 nM OHT for 48 hours and then allowed to grow in culture for 5 days from the onset of OHT treatment: subsequently all clones were analyzed by flow cytometry. 27Flip parental cells were used as a negative control. X-axis, mCherry fluorescence, Y-axis, cell number.

In a previous study [24] an OHT concentration of 50 nM was used to induce mCrem mediated recombination, significantly lower than the 1 µM used in the studies above. Thus, we wanted to investigate the interaction between mCrem expression level and OHT dose for induction of mCrem mediated recombination. Thus, we treated both the high and low expressing 27Flip/Cre clones with 100, 10, or 1 nM OHT for 48 hours, and compared the treatment groups for Cre activity at 5 days after the start of OHT treatment (Fig. 4). Since during OHT treatment the construct spends equal time in the forward and reverse orientations, all cells express mCherry during this time: as such, we included a “rest” period of 3+ days to allow white cells to lose any residual fluorescence. Clones 15 and 20 with constitutive flipping activity exhibited only minor changes in population distribution after OHT treatment - these minor changes may reflect differing efficiencies of flipping in the forward and reverse directions or differential effects of tamoxifen toxicity on the two populations at the higher doses. Clones with lower mCrem expression all exhibited dose dependent recombination, with the clone with the lowest apparent mCrem expression (based on a qualitative assessment of the western blot in Fig. 3A), 27Flip/Cre31, showing the lowest level of activity at 1 nM Cre. This result suggests that at sufficiently low OHT concentration, mCrem expression level becomes limiting for recombination efficiency. Nevertheless, although mCrem expression in this clone was near the limit of detection in our western blot, we saw flipping in 100% of cells at 10 nM OHT, suggesting that this concentration is sufficient to maximize Cre activity when applied for 48 hours. Consistent with previous work [11] we observed significant mCrem mediated toxicity at 1 µM and 100 nM OHT, which decreased at 10 nM and was essentially absent at 1 nM. In addition, the frequency of cell death was consistently higher among high mCrem expressors as compared to low expressors at 100 and 10 nm OHT, plateauing at 1 µM and being undetectable at 1 nM for all clones (data not shown).

### Efficiency of OHT-dependent Cre activity correlates with duration of OHT exposure

**Figure 5:**
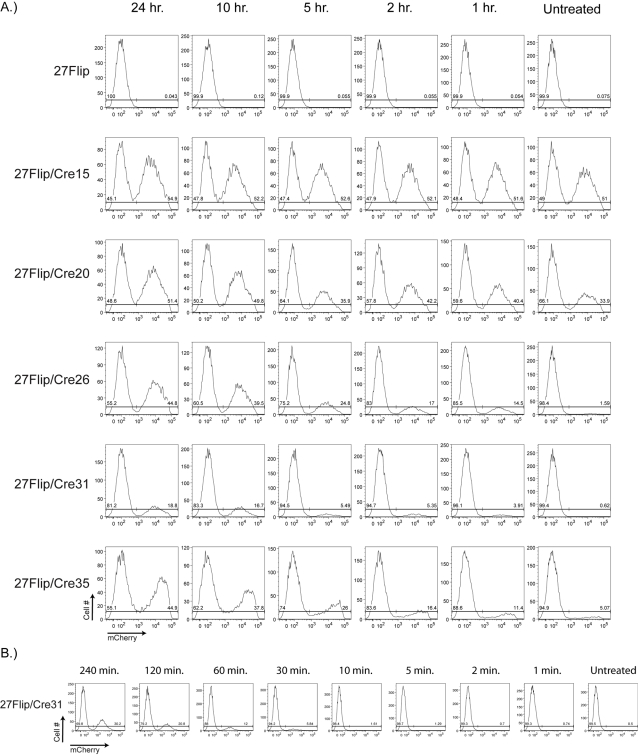
Efficiency of OHT-dependent flipping of the floxed cassette correlates with duration of OHT exposure in low mCrem expressors. *A* 27Flip/Cre clones were either left untreated or treated with 1 nM OHT for 24, 10, 5, 2, or 1 hours, and then allowed to grow in culture for 5 days: subsequently all clones were analyzed by flow cytometry. 27Flip parental cells were used as a negative control. *B* 27Flip/Cre31 cells were either left untreated or treated with 100 nM OHT for 1, 2, 5, 10, 30, 60, 120, or 240 minutes and then allowed to grow in culture for 5 days: subsequently all clones were analyzed by flow cytometry. X-axis, mCherry fluorescence, Y-axis, cell number.

We noted that at all concentrations of OHT, the majority of treated cells became positive for red fluorescence within 24 hours (data not shown), suggesting that the majority of cells may already have initiated switching well before the 48 hours of OHT exposure typically employed by our and other labs [24]. We therefore set out to identify the effect of varying OHT exposure time in clones expressing different amounts of mCrem. We treated the 27Flip/Cre clones with 1 nM OHT for 1, 2, 5, 10, and 24 hours before removing OHT and allowing the cells to grow for five days in culture, at which point we examined the cells for red fluorescence by FACS (Fig. 5A). In all clones except the high expressor 27Flip/Cre15, a steady decline in recombination activity was evident as OHT exposure time decreased: for 27Flip/Cre15 flipping was already at ∼100% in the untreated population. Coupled with the dose response of the various clones, these results also provided further support for our conclusion from Fig. 3 that recombination efficiency decreases with decreasing mCrem expression. Flipping in 100% of the cells in all clones except 27Flip/Cre15 was not seen at exposure times less than 48 hours.

Surprisingly, we observed low level induction of Cre activity at even 1 hour of 1 nM OHT exposure, a much shorter time than is typically used for mCrem induction. Consequently we were curious if, by using a high concentration of mCrem we might be able increase the percentage of cells with Cre activity at early time points. Since we showed that 100% of 27Flip/Cre31 cells underwent flipping at 10 nM applied for 48 hours (Fig. 4), we reasoned that a ten fold excess of this saturating concentration would be sufficient to induce rapid Cre activity. Thus we applied 100 nM OHT to 27Flip/Cre31 cells for short periods of time, allowed the cells to grow in culture for 5 days, and then analyzed flipping by FACS (Fig. 5B). Under these conditions, we detected roughly 50% of cells undergoing flipping after 4 hours of treatment with 100 nM OHT, and that roughly 1% of cells had undergone flipping within just 10 minutes of OHT treatment. These results suggest that although high concentration/short time exposure of OHT can induce detectable recombination, recombination does not occur with any degree of synchronicity in DT40 cells, even within cells of a clonal population.

## Discussion

Although some studies of the mCrem system [20], [24], [36] have suggested that mCrem mediated recombination is tightly controlled in the absence of ligand, work from other labs suggested ligand independent mCrem mediated recombination might occur, albeit at low frequency [11], [19], [26], [27]. This discrepancy has not been systematically addressed to date *in vitro* or *in vivo*. This prompted us to use a reversible switching system for analysis of the parameters governing tamoxifen/OHT-regulated mCrem recombination of a genomic target. In this report, we show that robust mCrem expression correlates with a high level of tamoxifen/OHT-independent Cre activity, while clones expressing mCrem at the limit of western blot detection exhibit extremely tight regulation. Additionally, we demonstrate that the Tamoxifen/OHT dose response of clones varies with mCrem expression level.

In contrast to our results, it has been previously shown that in DT40 cells expressing a reversible switching construct and mCrem, little to no OHT-independent Cre activity occurred [24]. These differences may be due to epigenetic differences at the respective integration loci [37] or possibly the serendipitous use of a low mCrem expressing clone (Cre expression levels were not examined in those studies). Although it is difficult to predict or control the former, our results clearly show that limiting mCrem expression may be effective in controlling tamoxifen/OHT-independent mCrem mediated recombination, although at the expense of potentially requiring higher dose or duration of OHT treatment to induce maximal recombination. Thus, future studies using reversible switching should evaluate clones with a range of low but detectable mCrem expression to identify those which minimize unregulated mCrem activity but provide maximal recombination at the desired OHT does and time of exposure. Furthermore, it is important to monitor the stability of both the forward and reverse populations in any reversible switching study (particularly if an antibiotic resistance cassette is integrated in either direction, since this may mask switching out of the given orientation), for evidence of OHT-independent mCrem mediated recombination. If unlicensed Cre activity is an issue, clones that appear mCrem negative by western blot should be screened via FACS with or without OHT treatment, since such clones may express mCrem at levels low enough to be undetectable by western blot but nevertheless be capable, as our results suggest, of mediating mCrem dependent recombination.

Given that we observed tamoxifen/OHT-independent Cre activity in several high expressing clones, we speculate that this phenomenon is an intrinsic property of mCrem in DT40 cells, possibly because of their small cytoplasmic volume, which may encourage mCrem nuclear translocation as expression increases. It is probably further generalizable that higher expression levels of mCrem in any cell line or *in vivo* model will lead to a greater risk for unlicensed activity: previous findings suggest that tamoxifen/OHT-independent Cre activity also occurs in mice and murine cells cultured *in vitro* [26], [27]. Thus, our results suggest that investigators should limit mCrem expression in any Cre-loxp based model system where tight ligand-dependent regulation of mCrem activity is crucial (our results in Figs. 3B, and 4 indicate that even minimal expression of mCrem is sufficient for 100% penetrance of Cre activity given an adequate OHT dose and time of exposure).

Previous studies have examined the effects of tamoxifen/OHT dosage on inducible Cre-mediated recombination, but never in the context of variable Cre expression. Thus, our results provide insight on tamoxifen/OHT dose and/or time of exposure adjustments that must be made at varying Cre expression levels. If low mCrem expressing clones are selected for study, as we recommend above, investigators can expect that a sufficiently high dose of OHT must be applied if 100% penetrance of recombination is desired. Surprisingly, in our system a “sufficiently high dose” titrated to only 10 nM OHT applied for 48 hours for our lowest mCrem expressing clone, a dose 100 fold lower than our standard OHT dose and 5 fold lower than the dose used in a previous study [24]. Since work from our lab and others suggests that mCrem mediated toxicity correlates with OHT dose, we recommend the use of 10 nM OHT for 48 hours to maximize penetrance while minimizing toxicity. This recommendation is supported by our time course studies, since even in the highly expressing clone 27Flip/Cre20, 1 nM OHT exposure times of 24 hours or less resulted in decreased Cre activity. Nevertheless, exposure time and/or OHT dose can be adjusted considerably depending on mCrem expression and the desired level of penetrance. The finding that Cre activity is evident in some 27Flip/Cre31 cells within 10 minutes of high dose OHT (100 nM) application, but that activity in 100% of cells cannot be achieved within 4 hours was surprising, as it suggests that induction of mCrem activity within even a clonal cell population is highly asynchronous. Whether better synchronicity can be achieved by examining only cells at a similar point in the cell cycle or by applying toxic doses of ligand for short periods of time are interesting areas for future investigation.

In conclusion, we show for the first time that robust mCrem expression allows Cre activity independent of ligand binding, while low mCrem expressing clones are largely free of this effect, and that tamoxifen/OHT dose response correlates with changing mCrem expression. Time and dose-dependent effects on mCrem activity suggest limitations on the use of conditional targeting approaches for applications which require tight temporal coordination of Cre action within a cell population.

## Materials and Methods

### Cell Culture

DT-40 B lymphocytes were cultured at 37°C with 5% CO_2_ in RPMI 1640 Medium (Mediatech Inc.) supplemented with 10% fetal bovine serum (FBS; Mediatech Inc.), 2% chicken serum (Invitrogen), 10 units/ml penicillin/streptomycin (Mediatech Inc.), 2 mM glutamine (Mediatech Inc.) and beta mercaptoethanol (50 µM; Sigma).

### Molecular Biology

The “Flip” Construct backbone containing the loxp sites, 2 MCS sites, and the SV40 and BGH poly-A signals was synthesized by DNA 2.0, Inc. Subsequently, the CMV promoter, chicken NUDT9 cDNA, mCherry cDNA, and a B-actin-Neomycin resistance cassette were inserted using various restriction sites. The Left and Right homology arms extended from exon 4 to exons 1 and 7 respectively. Integration of the Flip construct into the NUDT9 locus was verified using the following primers, which span the Neo-Right homology Arm and the Right homology arm-genomic locus junctions in their product: Forward: ACATAGCGTTGGCTACCCGTGATA, Reverse: ACTGCTTTGAAGGCCACACATTCC. The product of this PCR was also sequenced following gel- or PCR purification. Following transfection, clones were selected in G418 (2 mg/mL, invitrogen), and selected clones were screened for targeted integration via PCR.

Mer-Cre-mer, expressed in the pcDNA5T/O vector (invitrogen), was a generous gift from Michael Reth by way of Tomo Kurosaki. Following transfection of mer-Cre-mer, clones were selected in hygromycin (2 mg/mL, Calbiochem), and selected clones were evaluated for mer-Cre-mer expression by western blot. Clones were lysed and 50 µg of protein from each clone were run on an 8% SDS-PAGE gel. Mer-Cre-mer was visualized using polyclonal rabbit anti-Cre antibody (1∶2000, Novagen, Inc.). β-actin controls were stained with a mouse polyclonal anti-β actin antibody (1∶40,000, Sigma). The secondary antibody was peroxidase conjugated donkey anti-rabbit from Amersham Pharmaceuticals.

Stable tranfection of DT-40 B lymphocytes was carried out using a Bio-Rad Gene-Pulser electroporation apparatus. Cells (1×10^7^/0.5 ml serum-free medium) were pulsed in 0.4-cm cuvettes with 50 µg plasmid DNA at 550 V and 25 µF.

### Flow Cytometry

FACS analyses were performed on either a BD FACSAria (Becton-Dickenson) or a BD LSRII (Becton-Dickenson), using a green laser (561 nm) to excite the mCherry protein (excitation max: 587 nm, emission max: 610 nm). Cells were resuspended in media as described above, without the color indicator. 10,000 events were collected for each panel. Sorting experiments were carried out on the BD FACSAria.

### Cre induction

Cells expressing mer-Cre-mer were induced using 1 µM, 100 nM, 10 nM, or 1 nM 4-Hydroxytamoxifen (OHT) for 48, 24, 10, 5, 2, or 1 hour, or left untreated. Following tamoxifen treatment, cells were centrifuged at 500× *g* for 5 minutes to collect the cells, resuspended in 10 mL RPMI, and centrifuged again to wash the cells. The cells were then resuspended in standard medium and grown in culture for 3–50 days.
